# Current status of catabolic, anabolic and inflammatory biomarkers associated with structural and symptomatic changes in the chronic phase of post-traumatic knee osteoarthritis– a systematic review

**DOI:** 10.1016/j.ocarto.2023.100412

**Published:** 2023-10-05

**Authors:** Oliver O'Sullivan, Peter Ladlow, Kat Steiner, Charles Hillman, Joanne Stocks, Alexander N. Bennett, Ana M. Valdes, Stefan Kluzek

**Affiliations:** a Academic Department of Military Rehabilitation (ADMR), Defence Medical Rehabilitation Centre (DMRC), Stanford Hall, Loughborough, UK; b Academic Unit of Injury, Recovery and Inflammation Sciences, Faculty of Medicine and Health Sciences, University of Nottingham, Nottingham, UK; c Department of Health, University of Bath, Bath, UK; d Bodleian Health Care Libraries, University of Oxford, Oxford, UK; e Nottingham University Hospitals NHS Trust, Nottingham, UK; f National Heart and Lung Institute, Imperial College London, London, UK; g Nottingham NIHR Biomedical Research Centre, Faculty of Medicine and Health Sciences, University of Nottingham, Nottingham, UK; h Department of Twin Research & Genetic Epidemiology, King's College London, London, UK; i Centre for Sport, Exercise and Osteoarthritis Research Versus Arthritis, University of Nottingham, Nottingham, UK

**Keywords:** Post-traumatic osteoarthritis, Biomarkers, Serum, Synovial fluid, Pathophysiology

## Abstract

Post-traumatic OA (PTOA) can occur within 5 years after a significant injury and is a valuable paradigm for identifying biomarkers. This systematic review aims to summarise published literature in human studies on the associations of known serum and synovial fluid biomarkers at least a year from injury to structural, symptomatic changes and underlying PTOA processes.

A systematic review was performed using PRISMA guidelines, prospectively registered on PROSPERO (CRD42022371838), for all ‘wet’ biomarkers a year or more post-injury in 18–45-year-old participants. Three independent reviewers screened search results, extracted data, and performed risk of bias assessments (Newcastle-Ottawa Scale). Study heterogeneity meant a narrative synthesis was undertaken, utilising SWiM guidelines.

952 studies were identified, 664 remaining after deduplication. Following first-round screening, 53 studies underwent second-round screening against pre-determined criteria. Eight studies, with 879 participants (49 ​% male), were included, measuring serum (n ​= ​7), synovial fluid (SF, n ​= ​6), or both (n ​= ​5). The pooled participant mean age was 29.1 (±4). 51 biomarkers were studied (serum ​= ​38, SF ​= ​13), with no correlation between paired serum and SF samples. One serum biomarker, cartilage oligomeric matrix protein (COMP), and four SF biomarkers, interleukin (IL)-1β, IL-6, tumour necrosis factor (TNF), and COMP, were measured in multiple studies.

Associations were described between 11 biomarkers related to catabolism (n ​= ​4), anabolism (n ​= ​2), inflammation (n ​= ​4) and non-coding RNA (n ​= ​1), with OA imaging changes (X-ray and MRI), pain, quality of life and function. Widespread differences in study design and methodology prevented meta-analysis, and evidence was generally weak. A unified approach is required before widespread research and clinical biomarker use.

## 1 Background

Osteoarthritis (OA), the most common form of arthritis with a rising incidence and prevalence globally [1], is a heterogeneous, progressive joint disease associated with changes to the synovium, cartilage and bone, leading to pain, stiffness, loss of function and increased inactivity [2]. Typically, OA takes years to develop, modulated by the interaction of physical, immunological and mechanical factors, with an asymptomatic and pre-radiographic molecular phase prior to radiographic and symptomatic phases [3]. However, the sub-type post-traumatic OA (PTOA), has an accelerated pathological process, with symptoms appearing a few years after injury [4].

Due to the distinct initiating event leading to PTOA, it is of interest to researchers, with a specific focus on molecular diagnostics through the use of biological markers (biomarkers) to understand pathological pathways prior to joint dysfunction, allowing earlier identification and intervention [[3], [4], [5], [6]]. Biomarkers have a wide range of potential applications, including for disease-modifying OA drug (DMOAD) trials, for the targeted identification and recruitment of those with high-risk progressive OA and as outcome measures alongside existing measures including joint space narrowing and pain scores [6,7]. In addition, clinical use of biomarkers will allow early, pre-symptomatic identification of disease activity, allow commencement of preventative measures, and demonstrate the effect of interventions on the post-traumatic development of OA [8].

Any proposed biomarker is only as informative as its ability to quantify the pathophysiological change it is supposed to represent. PTOA development follows disturbed joint homeostasis, as a result of changes in joint loading triggered by pain or structural changes, or alterations in the production of inflammatory mediators, growth factors, and extracellular matrix components, with the suggestion that structural changes influence local bone and cartilage compositional changes [[9], [10], [11], [12], [13]]. Those processes, and resultant imbalance between anabolic and catabolic pathways, are likely to represent a failure of initial injury repair and/or remodelling, and involve the generation of new, and adaptation of existing, tissue, including cartilage matrix macromolecule synthesis or subchondral bone resorption, mediated by cytokines [14]. They can be monitored with established biomarkers, including cartilage-derived markers such as cartilage oligomeric matrix protein (COMP), a marker of cartilage metabolism, seen in OA to predict osteoarthritic bony and cartilage changes [[15], [16], [17]] and identified in the early stages of PTOA [18], and pro-inflammatory markers such as interleukin-1β (IL-1β), with increased levels mediating prolonged inflammation and activating chondrocyte catabolism in OA [19], seen in significantly raised concentrations following traumatic injury [20].

Current DMOAD studies include those investigating established pharmacological agents and those identifying exploratory modalities such as mesenchymal stem cell-derived exosomes [6,[21], [22], [23]]. A panel of molecular biomarkers has been proposed by the Federal Drugs Administration/Osteoarthritis Research Society International (FDA/OARSI) initiative for drug discovery and development, with many other biomarkers in the experimental stage [6,22]. However, these biomarkers have not yet been fully approved, with further work required to understand the relationships of biomarkers to underlying pathophysiology and individual patient phenotypes. This would allow biomarkers to guide phenotype-specific interventions and judge the impact of specific pharmacological treatments on pre-identified pathophysiological processes in a very heterogeneous condition [8,24,25]. Examples of this include the use of intra-articular steroid to suppress inflammation as an anti-catabolic agent [24], or Sprifermin, recombinant human fibroblast growth factor-18, as an anabolic agent [26].

Previous reviews of knee PTOA biomarkers have sought to understand those implicated in acute (hours-day) and post-acute (days-weeks) phases following injury [12,18,27,28]. Our recent review of chronic imaging biomarkers revealed a correlation between structural alterations of the knee joint after injury with markers of cartilage and bone composition, as well as clinical outcomes, suggesting a link to the underlying pathophysiological processes [13]. The hypothesis of this systematic review is that serum and synovial fluid biomarkers remain elevated into the chronic phase and are linked to structural and patient-reported outcomes, potentially offering insights into PTOA mechanisms. Therefore, this systematic review aims to summarise published literature in human studies on the associations of known serum and synovial fluid biomarkers at least a year from injury to structural and symptomatic changes and underlying PTOA processes.

## 2 Methodology

A systematic review was conducted in line with PRISMA guidance [29]. Inclusion criteria included full-text studies in languages spoken by the research team, in participants with a significant knee injury aged 18–45 (to avoid confounding with skeletal immaturity or idiopathic OA), involving ‘wet’ biomarkers measured at least a year from injury (to ensure physiological remodelling changes have concluded) (Table 1). The protocol was registered prospectively on PROSPERO (CRD42022371838).

**Table 1:** Study selection inclusion and exclusion criteria.

Inclusion criteria	Exclusion criteria
Full text articles, in English, Polish, Danish, or Spanish	Laboratory-based, in-vivo or animal studies
Participants aged between, inclusive of, 18 and 45 years old	Participants under 18 or over 45 years old
Significant injury one year or more previously	Significant injury sustained less than 1 year ago
Study involved ‘wet’ biomarker (including serum, plasma, urine or synovial fluid)	

Medline and Embase (both via Ovid), Cochrane CENTRAL (via Wiley) and ClinicalTrials.gov were all searched on 8/11/22, and WHO ICTRP on 9/11/22. Conference proceedings were searched on 10/11/22. Corresponding authors of similar systematic reviews registered on PROSPERO were contacted. Subject matter experts recommended additional studies in addition to those found in searches. A hedge for human studies was used in Medline and Embase [30]. No other filters or limits were used. Searches incorporated keywords and subject headings relating to knee PTOA and biomarkers. The full search strategy can be found in Supplementary File 1. Results were deduplicated using EndNote 20 and SR Accelerator.

Initial title and abstract screening were performed by two reviewers independently against pre-determined eligibility criteria (Table 1) with a third reviewer resolving conflicts, using Rayyan (www.rayyan.ai). A second full-text screen, and subsequent data extraction, were undertaken in the same manner with the same reviewers. Data extraction was performed using a pre-prepared data extraction form (Excel, Microsoft). Data extracted included;

- First Author, Title, Journal, Year
- Population: Number (cases/control), Sex, Injury Type, Occupation (if mentioned)
- Biomarkers: Which Used, Type, When/How Measured, What Comparator

Risk of bias assessment was performed using the Newcastle-Ottawa Scale (NOS) assessment tool by two reviewers independently [31].

Due to the heterogeneity of studies, direct comparison and meta-analysis were not possible, so a narrative review was undertaken in line with the Synthesis without Meta-analysis (SWiM) guidelines (Supplementary File 2) [32].

## 3 Results

**Fig. 1:**
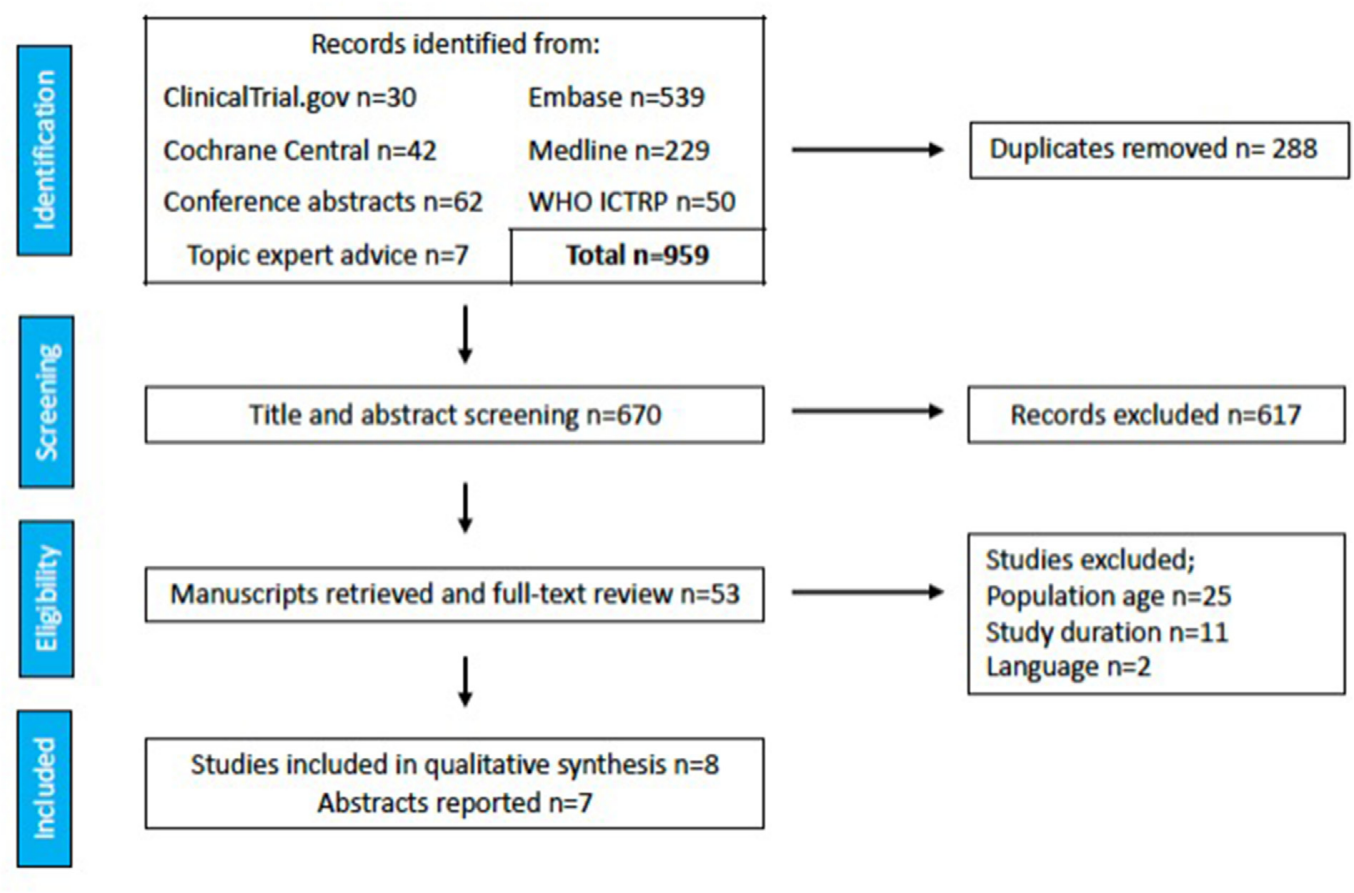
PRISMA flow diagram of systematic review.

A total of 952 studies were identified following the initial search. Following deduplication, 664 articles remained. A title/abstract screen was performed, identifying 53 papers meeting eligibility criteria. Manuscript full text for all these were sought and retrieved. Following full-text screen, 8 studies met the criteria for inclusion [[33], [34], [35], [36], [37], [38], [39], [40]]. In addition, 7 conference abstracts met inclusion criteria and, in line with Cochrane recommendations, are reported in Supplementary File 3 [[41], [42], [43], [44], [45], [46], [47], [48]]. Excluded studies can be found in Supplementary File 4; the most common reasons for exclusion were time from injury to biomarker measurement and participant age. Fig. 1 displays the PRISMA diagram.

In total, 879 participants were included in the eight selected papers, 634 injured (72 ​%) and 245 comparison participants (28 ​%). However, three studies use data from the Knee Anterior Cruciate Ligament, Nonsurgical versus Surgical (KANON) study [36,37,39], so there are 650 individual participants involved (injured n ​= ​405, 62 ​%).

The pooled mean age of participants was 29.1 (±4), excluding a single study where participant age was only reported as ‘less than 41’ [33]. Female sex confers an increased risk of PTOA [49], and was recorded in all studies bar one [33]; 26 ​% [36,37,39], 44–45 ​% [34,38], and 84 ​% female [35] respectively, with one study only recruiting male participants [40](Table 2). Across all studies, 51 ​% of participants were female. There were no restrictions on ethnicity, only one study reported the ethnic origin of their population (Chinese) [38]. One study involved individuals with unilateral lower-limb amputation, sustained in combat-injury [40], with the remainder sustaining anterior cruciate ligament (ACL) injury. Two studies report injury aetiology, military combat [40], and during sport (86 ​%) and everyday activity (14 ​%) [38]. Study sample sizes ranged from n ​= ​11 [34] to n ​= ​121 [36]. All studies used cross-sectional methodology to measure association.

**Table 2:** Study characteristics of included studies.

Author, year	n ​= ​case/control Age, mean (SD) Sex, M:F	Type (s/sf)	Time from injury Years, mean (SD)	Markers measured	Imaging	PROMs	Clinical	Surgical/Histology
Zhang, 2012 [33]	n ​= ​102/60 <41YO^a^ sex *NR*^a^	s	1 post ACL-R	miRNA, snoRNA (U24, U38, U48, U49)	WORMS (MRI)	–	–	–
Ahlen, 2015 [34]	n ​= ​11/0 26.1 [7] 6 ​M:5F	sf	8 post ACL-R (2–48 ​m from inj to ACL-R)	IL-1β, IL-6, TNF-α, sGAG, ARGS-aggrecan, COMP	Presence/integrity ACL/PCL, meniscus, & cartilage (MRI) Fairbank (XR)	TAS, KOOS, Lysholm	Single-leg hop, pivot-shift, ROM, Lachman	–
Zou, 2016 [35]	n ​= ​61/65 30.5 [6]/31.1 [6] 10 ​M:51F/9 ​M:56F	s sf	6 (range 1–14)	sGherlin, sfGherlin, (IL-6, TNF-α, COMP, CTX-II)^b^	Reicher (MRI)	IKDC, Lysholm	–	Noyes scale, Mankin score
Struglics, 2018 [36]	n ​= ​121/50 (25sf/25s) 26 [5]/30 [12] sf & 31 [10] s 91 ​M:30F/16 ​M:9F sf & 13 ​M:12 ​F ​s	s sf	1 (n ​= ​64) 2 (n ​= ​121) 5 (n ​= ​121)	sCOMP, sfCOMP (Two immunoassays used, AnaMar (COMP-Ana) and BioVender (COMP-Bio)	–	–	–	–
Roemer, 2019 [37]	n ​= ​113/0 26 [5] 85 ​M:28F	s sf	2 5	sIL-6/8/10/12p70, sTNF-α, sIFN-γ, sfIL6/8/10, sfTNF-α, sfIFN-γ	ACLOAS (MRI) Kellgren-Lawrence (XR)		–	–
Sun, 2019 [38]	n ​= ​72/70 30 [6]/30 [5] 40 ​M:32F/36 ​M:24F^c^	s sf	9 (range 6–16)	sPACAP, sfPACAP, (IL-1β, TNF-α)^b^	Reicher (MRI)	VAS, IKDC, Lysholm	–	Mankin score
Struglics, 2020 [39]	n ​= ​116/0 28 [5] 86 ​M:30F	s sf	2	sIL-6/8/10/12p70, sTNF, sIFN-g116, sfIL6/8/10, sfTNF,	ACLOAS (MRI)		–	–
Wasser, 2022 [40]	n ​= ​38/0 37 [7] 38 ​M:0F	s	10 (7)	CTX-1, HA, C2C, PIIANP, NTX-1, CCL-2/4/5/11, CXCL, COMP, IFN-α, IL-1α/7, SDF-1, TIMP-1, TNF-α, MMP-2/3/7/8/9/12/13	Outerbridge (MRI) Kellgren-Lawrence (XR)	VAS, KOOS, VR-36, SF-8	15 ​m gait assessment	–

SD: Standard Deviation, M: Male, F: Female, PROMs: Patient Reported Outcome Measures, s: serum, sf: synovial fluid, YO: Year Old, NR: Non Reported, ACL: Anterior Cruciate Ligament, ACL-R: ACL Reconstruction, OA: Osteoarthritis, miRNA: Micro Ribonucleic acid, snoRNA: small nucleolar RNA, MRI: Magnetic Resonance Imaging, WORMS: Whole Organ MRI score, XR: X-ray, PCL: Posterior Cruciate Ligament, TAS: Tegner activity scale, KOOS: knee injury and OA outcome score, IKDC: international knee documentation committee, ROM: Range of movement, VR-36: Veterans-RAND, SF-8: Short Form 8, IL: interleukin, sGAG: sulphated glycosaminoglycans, COMP: cartilage oligomeric matrix protein, TNF: tumour necrosis factor, CTX: collagen cross-linked C-telopeptide, IFN: interferon, PACAP: pituitary adenylate cyclase activating polypeptide, HA: hyaluronic acid, C2C: Cleavage of Type II collagen, NTX: N-telopeptide of Type 1 Collagen, PIIANP: N-Propeptide of Collagen IIA, TIMP: Tissue inhibitor matrix metalloproteinase, SDF: Stromal cell-derived factor, MMP: Matrix metalloproteinase, CCL: Chemokine (C–C Motif) ligand, CXCL: Chemokine (C-X-C Motif) Ligand, K-L: Kellgren-Lawrence, OC: Outerbridge.

a Study authors were contacted.

b It is not reported if these markers were in serum as well as synovial fluid.

c The control population is described ambiguously.

**Fig. 2:**
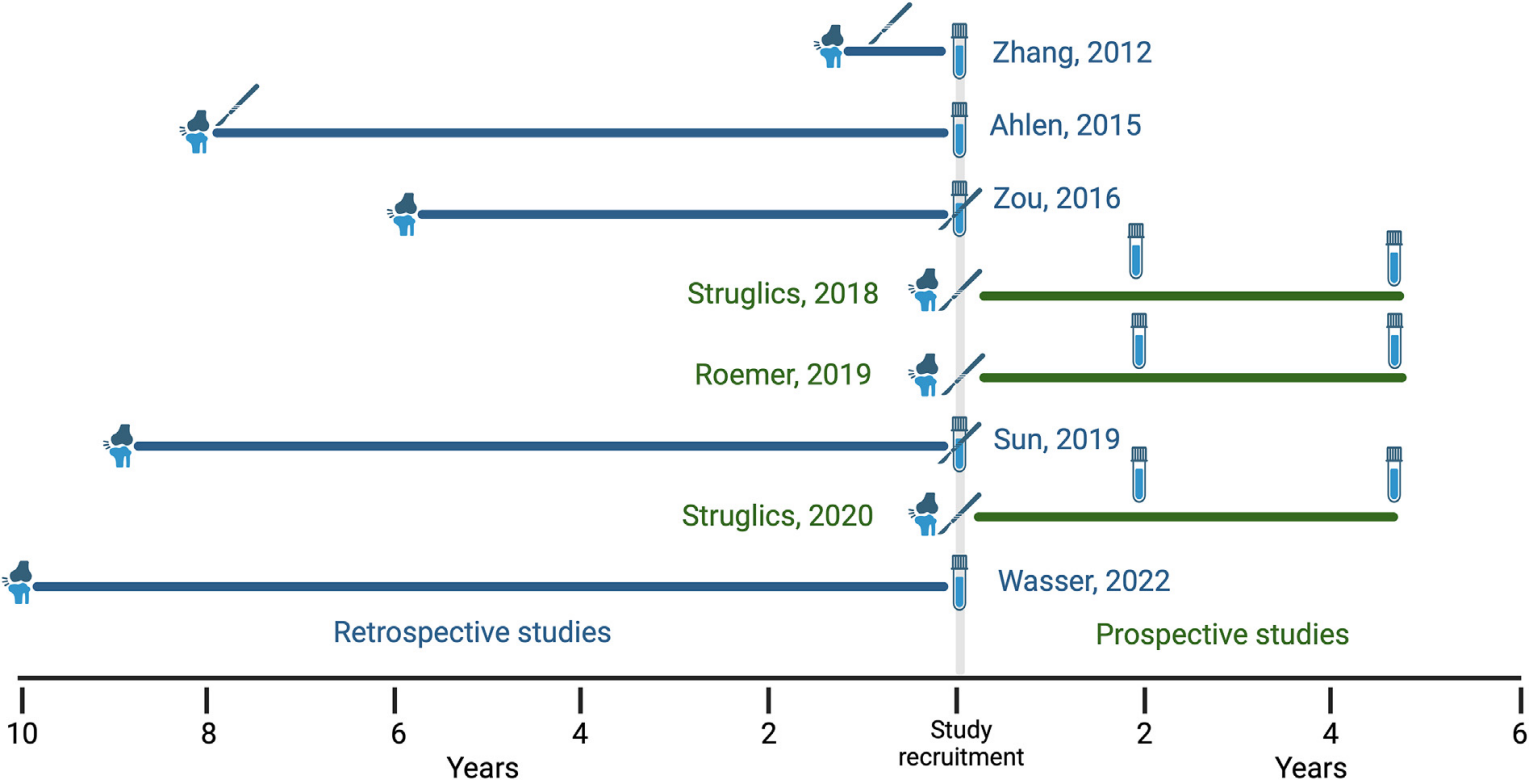
Pictorial representation of study designs, including time from injury and surgery (when reported), sample collection and direction of study. Blue colour represent retrospective studies, green colour represent prospective studies. Knee icon represents time of initial injury (when reported), scalpel icon represents time of surgery (when reported) and sample tubes represent data collection points. Created in BioRender. (For interpretation of the references to color in this figure legend, the reader is referred to the Web version of this article.)

Studies varied in terms of design and observation periods. Four studies had a comparison population, only one of which matched the exposed population [35]. One study comparison was half age-matched ‘within 7 years’ and the rest older [33]. One study used one reference population to compare serum and another for synovial fluid [36], and the last did not fully describe their reference population [38]. Another study used the contralateral limb as a reference [34]. Studies ranged from a mean of one to 10 years post-injury, with some individuals 14-[35], 16-[38], and 18-years [40] from their initial traumatic injury (Fig. 2). The date from injury was confounded by some studies, with two reporting the date from ACL reconstruction (ACL-R) operation (the latter did also report average time from injury to ACL-R) [33,34]. All studies measured serum or synovial fluid, with only samples taken at least a year from injury included in this review. No studies involving plasma-, urinary-based biomarkers or metabolomics were identified.

## 4 Dependent and independent variables

### 4.1 Dependent variables

Comparators used to measure biomarker associations included imaging, patient-reported outcome measures (PROMS), histopathology and/or biomechanical assessment (Table 2).

All, bar one, studies performed MRI (88 ​%), with five studies specifying 1.5 ​T magnetic field strength [33,34,36,37,39], employing a variety of scoring methods;

• The KANON studies used the ACL OA Score (ACLOAS) [36,37,39,50].
• One used the semi-quantitative whole organ MRI score (WORMS) [33,51].
• One study scored using the modified Outerbridge grading for MRI [[40], [52]]
• Two utilised the Reicher classification for meniscal tears [35,38,53].
• One study measured the presence and integrity of the cruciate ligaments, meniscus and cartilage [34].

Radiographical scoring also varied in the 30 ​% of studies measuring it;

• One study scored their weight-bearing XRs using Fairbanks [34,54].
• Two studies used Kellgren-Lawrence (K-L) on their weight-bearing radiographs, [55] with one utilising a K-L grade of ≥2 [37], and the other a K-L grade ≥1 [40], to define OA
• One study also measured using radio-anatomical positions and joint angles [40].

PROMs related to pain, function and quality of life, measured in 44 ​% of studies, included;

• Visual analogue scale for pain [38,40].
• Eight-item functional knee Lysholm scale [34,38,56].
• Knee injury and OA outcome score (KOOS), with five symptomatic and functional subscales [34,40,57].
• International knee documentation committee, utilising 18 questions related to symptoms [38].
• Veterans-RAND and Short form-8 for quality of life [40,58].
• Tegner activity scale to determine the level of sports participation [34].

Two studies included clinical and biomechanical assessments, including single-hop, pivot-shift, Lachman's and range of motion [34] and a 15 ​metre gait assessment [40]. In addition, two studies used histopathology scores (Noyes and Mankin) [35,38,59,60].

### 4.2 Independent variables

Seven studies (87.5 ​%) measured serum biomarkers [33,[35], [36], [37], [38], [39], [40]], six studies (75 ​%) measured synovial fluid biomarkers [[34], [35], [36], [37], [38], [39]], and five (62.5 ​%) measured both [[35], [36], [37], [38], [39]]. To assess correlation, all five studies involving paired serum and synovial fluid samples measured virtually the same panel (total exposed n ​= ​383).

All studies described sample collection (including centrifugation and freezing) and laboratory techniques. Two studies reported fasted serum sample collection [35,38]. Four synovial aspirations (80 ​%) were performed without lavage [[36], [37], [38], [39]]; one was performed under ultrasound by an experienced radiologist [34]. Two studies (40 ​%) had synovial fluid from a comparison group [36,38].

**Fig. 3:**
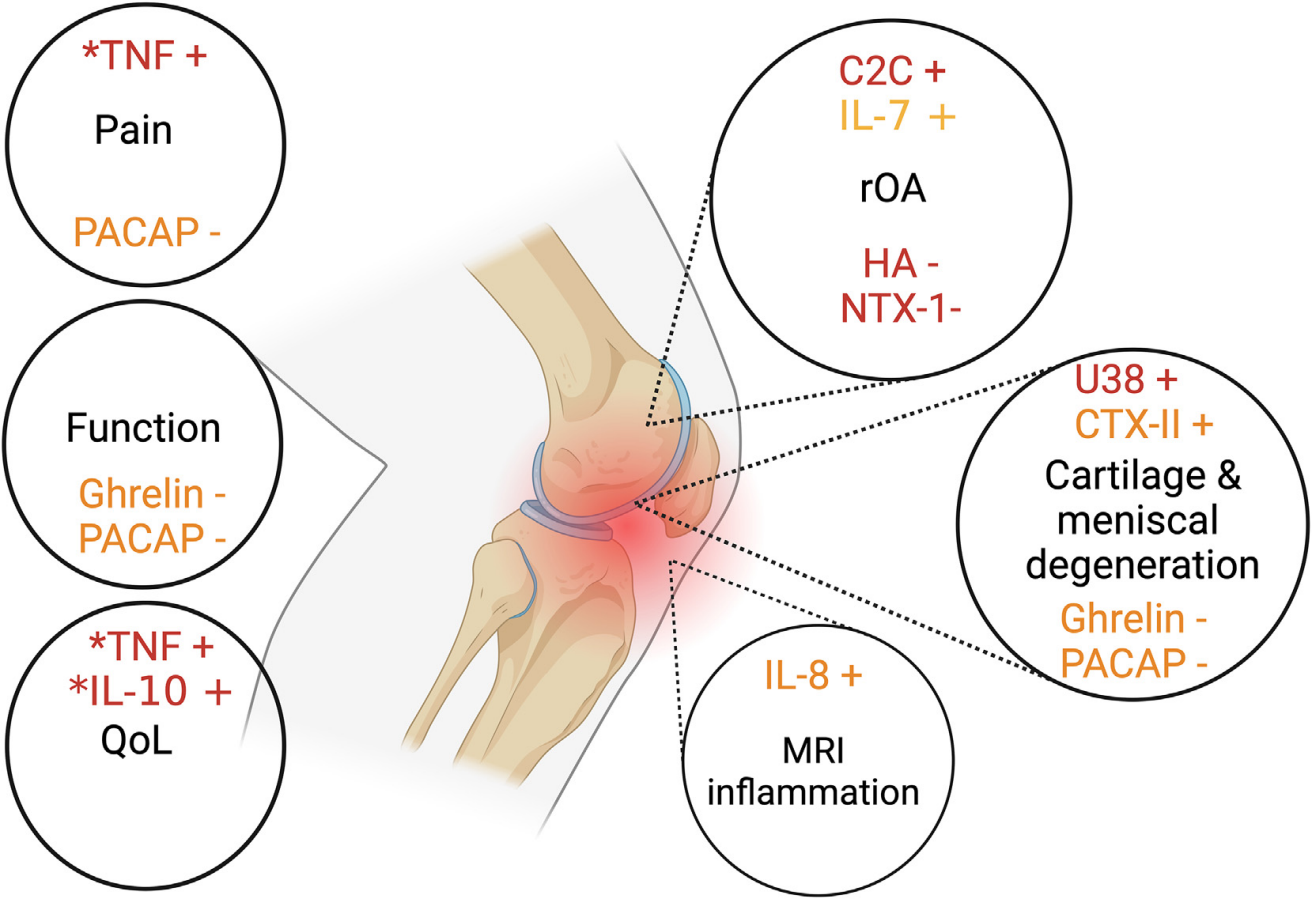
Associations identified between serum (red) and synovial fluid (orange) biomarkers to radiological and patient-reported outcome measures for post-traumatic osteoarthritis of the knee. TNF: Tumour necrosis factor, PACAP: Pituitary adenylate cyclase activating polypeptide, C2C: Cleavage of Type II collagen, IL: Interleukin, NTX: N-telopeptide of type 1 collagen, HA: Hyaluronic acid, CTX: Type II collagen cross-linked C-telopeptide rOA: Radiological osteoarthritis, MRI: Magnetic Resonance Imaging, QoL: Quality of life. ​+ ​indicates positive correlation – indicates negative correlation ∗biomarker concentration has undergone log^10^ transformation. Created in BioRender.

**Fig. 4:**
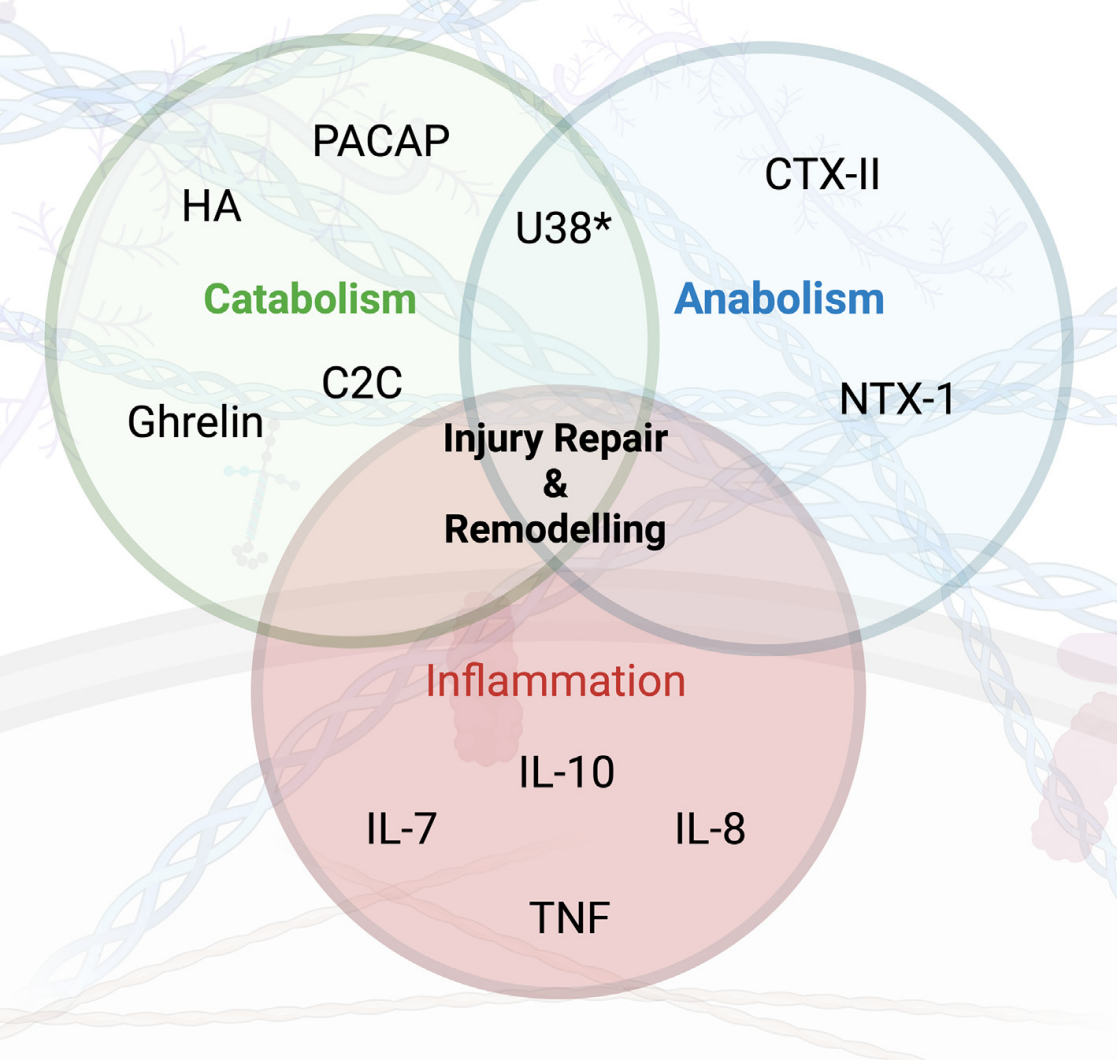
Summary of the main action of each biomarker of significance and their interaction during injury repair and remodelling. TNF: Tumour necrosis factor, PACAP: Pituitary adenylate cyclase activating polypeptide, C2C: Cleavage of Type II collagen, IL: Interleukin, NTX: N-telopeptide of type 1 collagen, HA: Hyaluronic acid, CTX: Type II collagen cross-linked C-telopeptide. ∗action of non-coding RNA U38 is uncertain. Created in BioRender.

A variety of markers were analysed (Table 2), with five performed by more than one study (Table 3). Three studies focussed on one marker, including ghrelin [35], COMP [36], and pituitary adenylate cyclase-activating polypeptide (PACAP) [38], with others assessing a panel of markers [33,37,39,40](Table 2). Three studies reported values in relevant units [35,36,40], two used log10 to calculate associations [37,39]. Most studies used either multiplex or enzyme-linked immunosorbent assay (ELISA), and a single study used reverse transcription and preamplification prior to polymerase chain reaction (PCR) [33]. Cross-sectional associations are described below, with biomarkers classified by their primary mechanism (catabolic, anabolic, inflammatory), and summarised in Fig. 3, Fig. 4, with a complete description of study results in Supplementary File 5.

**Table 3:** Serum and synovial fluid biomarkers performed by multiple studies.

Marker	Company of Assay used (mean time from injury)	Associations and/or correlations
Serum		
COMP^a^	AnaMar (5 ​yrs) [36] BioVender (5 ​yrs) [36] R&D Systems Inc (11 ​yrs) [40]	Positively correlated with age, increased in males, associated with other biomarkers [36], no difference between injured and controls [36,40]
Synovial fluid		
IL-1β	Meso Scale Discovery (8 ​yrs) [34] Cosmobio Co Ltd (9 ​yrs) [38]	No difference between injured and controls, [34] poor discrimination for meniscal injury [38]
IL-6	Meso Scale Discovery (8, 2, & 2 ​yrs) [34,37,39] IBL America (6 ​yrs) [35]	No difference between injured and controls, [34] no association with PROMs, [39] no association with inflammatory MRI biomarkers and weak discriminatory accuracy for knee OA in combined model [37], poor discrimination for meniscal injury [35]
TNF-α	Meso Scale Discovery [34,37] (8 & 2 ​yrs) IBL America [35] Cosmobio Co Ltd (9 ​yrs) [38]	No difference between injured and controls, [34] no association with inflammatory MRI biomarkers and weak discriminatory accuracy for knee OA in combined model, [37] poor discrimination for meniscal injury [35,38]
COMP	AnaMar Medical AB (8 ​yrs) [34] R&D Systems Inc (6 ​yrs) [35] BioVendor (5 ​yrs) [36]	No difference between injured and controls, [34] poor correlation to meniscal injury, [35] higher in males and injured cohort and associations with multiple other molecular biomarkers [36]

Note: Two studies completed the same panel in the same population, Roemer [37] and Struglics (2020) [39], so the markers that only they share are not included in this table.

COMP: cartilage oligomeric matrix protein, BMI: Body Mass Index, IL: interleukin, MRI: Magnetic Resonance Imaging, TNF: tumour necrosis factor, OA: Osteoarthritis, OARSI: Osteoarthritis Research Society International, FDA: Federal Drugs Administration.

a Serum COMP is on the OARSI FDA Osteoarthritis Biomarker Working Group panel [5].

#### 4.2.1 Serum markers

The total number of serum biomarkers measured across all studies was 38.

Serum biomarkers measured either anabolic, catabolic or pro-inflammatory processes (Table 2) [[35], [36], [37], [38], [39], [40]]. In addition, one study measured microRNA (miRNA) and small nucleolar RNA (snoRNA) [33].

### 4.3 Catabolic biomarkers

Serum biomarkers included in this review associated primarily with catabolism were hyaluronic acid (HA) [40], cleavage of type II collagen (C2C) [40], tissue inhibitor of metalloproteinase (TIMP)-1 [40], stromal cell-derived factor (SDF)-1 [40], N-propeptide of collagen IIA (PIIANP) [40], ghrelin [35] and PACAP [38].

HA levels were 73 ​% lower and C2C was 44 ​% higher in the injured group with radiographic OA compared to those without radiographic change in a study with wide variability in time from injury and minimal matching between groups. No other catabolic biomarkers demonstrated any relationships to dependent variables.

### 4.4 Anabolic biomarkers

Biomarkers related to anabolism included N-telopeptide of type 1 collagen (NTX-1), COMP (measured by three different assays, Table 3) [36,40], matrix metallopeptidase (MMP)-2/3/7/8/9/12/13 and type I collagen cross-linked C-telopeptide (CTX-1), all measured in the same study [40].

In those with radiographic OA, NTX-1 was 49 ​% lower compared to those without, in a study with wide variation in time from injury, and minimal between-group matching [40]. In one study, COMP showed no differences between groups [40]. In a second study, COMP did have a relationship with age, sex and other biomarkers, but not with injury, however, multiple imputation was used, which might have masked the associations at lower detection levels [36]. No other anabolic biomarkers demonstrated a relationship to dependent variables.

### 4.5 Inflammatory biomarkers

Inflammatory serum biomarkers included chemokine (C–C Motif) ligand (CCL)-2/4/5/11 [40], chemokine (C-X-C Motif) ligand (CXCL) [40], IL-1α[40], IL-6 [[37], [39]], IL-7[40] , IL-8 [[37], [39]], IL-10 [[37], [39]], IL-12p70 [[37], [39]], interferon (IFN)-γ[37], IFN-α[40], IFN-g116 [39], tumour necrosis factor (TNF) [39] and TNF-α[[37], [40]].

IL-7 had a 180 ​% higher concentration in those with radiographic OA compared to those without [40], with the same caveats as previously. TNF and IL-10 demonstrated a relationship to worsening KOOS scores (TNF to KOOS-pain, QoL and KOOS4, and IL-10 to QoL) in adjusted multivariable and unadjusted univariable linear regression, though this study did utilise multiple imputations and did not adjust for treatment (surgical vs. non-surgical) [39]. No other serum inflammatory biomarkers demonstrated a relationship with the dependent variables.

### 4.6 Non-coding RNA

One study measured non-coding RNA, using miRNA and snoRNA [33]. Serum snoRNA U38 concentrations were higher in those with significant cartilage degeneration (WORMS score ≥4), though this study was limited by an unclear methodology, significant results were only found on sub-group analysis, lack of correction for multiple testing and undetectable levels of snoRNA U38 in the control group [33]. Neither miRNAs nor the other snoRNAs showed any significant associations.

#### 4.6.1 Synovial fluid markers

Five studies collected synovial fluid samples in addition to serum samples [[35], [36], [37], [38], [39]], while the final study [34] collected only synovial fluid samples to measure the local effect of biomarkers. Markers of anabolism, catabolism and inflammation were measured (Table 2). The total number of synovial fluid biomarkers measured was 13.

### 4.7 Catabolic biomarkers

Synovial fluid biomarkers related to catabolism were ARGS-aggrecan [34], sulphated glycoaminoglycans (sGAG) [34], ghrelin [35], and PACAP [38].

Synovial fluid ghrelin and PACAP were both seen to be negatively correlated to histological severity and positively correlated to PROMs related to pain and function [35,38], although Zou [35] had a wide range of time from injury, no demographic data and no control synovial fluid samples, and Sun [38] had ambiguity regarding their control participants.

No other catabolic biomarkers demonstrated a relationship to the dependent variables.

### 4.8 Anabolic biomarkers

The synovial fluid biomarkers related to anabolism were CTX-II[35] and COMP[[34], [35], [36]].

CTX-II was seen to have an area under the curve (AUC) of >0.70 for meniscal injury, however, this study was missing participant demographic data and synovial fluid samples in the control group, with a wide variety of time from injury [35]. Three studies examined COMP. One showed that synovial fluid COMP showed no association to injury in the smallest study population with extensive variation in age and time from injury [34], in another COMP provided no predictive value for meniscal injury [35], and a third study demonstrated significantly higher concentrations of synovial fluid COMP in the injured cohort and was associated with other molecular biomarkers (including ARGS-aggrecan, NTX-1 and CTX-II), however, this study did not have synovial fluid samples for the entire study population [36].

### 4.9 Inflammatory biomarkers

Pro-inflammatory synovial fluid biomarkers included IL-1β[[34], [38]], IL-6 [[34], [35]], IL-8 [[37], [39]], IL-10 [[37], [39]], TNF[39], TNF-α[[34], [35], [37], [38]], and IFN-γ[37], with more homogeneity across studies studying the same markers.

IL-8 showed weak associations to MRI-related inflammation (specifically, grade 2/3 effusion-synovitis on WORMS) in an unadjusted model, in a study population missing some samples and requiring multiple imputation [37]. No other synovial fluid inflammatory biomarkers demonstrated a relationship to the dependent variables.

#### 4.9.1 Risk of bias assessment

Risk of bias assessments were performed for all studies using the NOS (Table 3). NOS measures three elements of the study design; selection of participants, comparability of cases and controls, and assessment and ascertainment of outcomes [31]. Studies are graded using a points system; unsatisfactory 0–4, satisfactory 5–6, good 7–8 and very good 9–10 points. The cross-sectional NOS proforma was used in all bar two - when the cohort proforma was more appropriate (maximum score 9). One study was rated unsatisfactory [33]; four satisfactory [[36], [37], [38], [39]]; and three good [34,35,40](Table 4).

**Table 4:** Risk of bias assessment using Newcastle Ottawa Scale.

Author, Date	Selection	Comparability	Outcome	Overall	Rating
Zhang, 2012	∗∗	–	∗	3	Unsatisfactory
Ahlen, 2015	∗∗∗∗	–	∗∗∗	7	Good
Zou, 2016	∗∗	∗∗	∗∗∗	7	Good
Struglics, 2018	∗∗∗	–	∗∗∗	6	Satisfactory
Roemer, 2019ˆ	∗∗	NA	∗∗∗	5	Satisfactory
Sun, 2019	∗∗	–	∗∗∗	5	Satisfactory
Struglics, 2020ˆ	∗∗	NA	∗∗∗	5	Satisfactory
Wasser, 2022	∗∗∗	∗∗	∗∗∗	8	Good

Cross-sectional study assessment version was used, except those marked with ˆ which used the cohort study version.

## 5 Discussion

This systematic review summarises the findings of eight studies, which analysed the serum and synovial fluid biomarkers from 405 separate exposed participants in the chronic phase after joint injury. Across all studies, 51 serum or synovial fluid biomarkers were studied, with 11 individual biomarkers related to catabolism, anabolism and inflammation seen to be associated with radiological changes (osteoarthritic, cartilage and inflammatory) or PROMs (pain, function, quality of life) (Fig. 3) at least a year from injury, and may offer an insight into a future biomarker panel, however, the strength of evidence is low due to study methodological weaknesses.

Classifying biomarkers based on their ability to monitor pathophysiological changes is fundamental for comprehending their value and utility in monitoring diverse processes in PTOA development [61]. The majority of markers studied in this review were associated with pro-inflammatory and catabolic processes (36%), with fewer measuring anabolic (18%) activities (Fig. 4). It is likely that the imbalance between the latter two processes leads to ineffective tissue repair or incomplete remodelling in a pro-inflammatory environment [2,14]. Equally, lowering pro-inflammatory mechanisms may lead to a decrease in cartilage repair and remodelling, accelerating cartilage deterioration, which may be observed in OA patients receiving steroid-based anti-inflammatory therapies. Further partitioning of outcome measures to monitor specific pathophysiological mechanisms, such as cartilage degeneration and development (e.g. COMP), osteophyte development (e.g. HA), or inflammation (e.g. IL-6) allows the understanding of responses to targeted mechanism-specific interventions. This is relevant for DMOAD development and would allow heterogenous study populations to be classified based on predominant pathophysiological pathways or likelihood of rapid progression, as well as overcoming some of the challenges associated with outcome measures [4,25,62,63].

Improving the understanding of pathway relationships is important (Fig. 4) [61]. Those associated with catabolism demonstrated some relationship with imaging, histology, and PROMs. Serum HA was lower, and C2C higher, in those with radiographic evidence of PTOA [40], with synovial fluid PACAP and ghrelin both positively associated with Lysholm and IKDC scores and negatively associated with VAS, MRI and histology [35,38]. Anabolic markers were seen to also be associated with imaging and structural change, with NTX-1 levels lower in those with radiographic OA and CTX-II consistently having an AUC of >0.70 for meniscal injury on MRI, exceeding the threshold for a clinically useful diagnostic test [35,64]. Many studies included COMP, which did not reveal a strong association with any dependent variable, and in fact, one study [36] characterised their negative serum COMP result as a “somewhat disheartening outcome” given the extensive use of the biomarker [65]. Variations of different ELISA kits and protocols for well-established markers may explain variations in the sensitivity, specificity, and accuracy of those findings, making it challenging to directly compare data from different studies and hindering the ability to establish universal cutoffs for abnormal cartilage turnover.

Inflammation is felt to be a key contributor to PTOA [19] and plays a role in direct response to injury, joint remodelling and adaptation in later stages. Across the pro-inflammatory biomarkers, there were relationships seen with IL-7 to radiographic OA [40], IL-8 with effusion-synovitis [37] and TNF with increased quality of life [39]. Effusion-synovitis has been seen acutely and chronically to demonstrate worse OA outcomes in those with traumatic joint injuries [13,66], and in a previous review, Khella reported synovial fluid TNF-α and IL-6 as ‘causal factors’ and IL-1β and IL-17 as ‘credible factors’ for PTOA progression [18]. This systematic review does not draw the same conclusion, suggesting further work is required to fully understand the interactions between tissue turnover and inflammation. The discrepancy in conclusions might be due to Khella's classification for the chronic phase (’1.5 months to years’), whereas this systematic review employed a more rigorous ‘one year or greater’.

Time from injury will likely influence biomarker concentration, depending on its source and role in ongoing joint pathology. All included studies had differing times from injury (Fig. 2), and this remains an important unanswered question requiring further attention, as do the relative change in biomarker concentration over time. Longitudinal studies, such as those cited in this review [37,39,40] and elsewhere [8], offer an opportunity for this.

The type of sample is relevant (Fig. 3). Serum biomarkers are well-studied, often due to ease of measurement, however, similar to previous reviews [12,61], there is currently no strong evidence to suggest any single serum biomarker can be used individually for diagnosis, prognosis, or to measure the impact of an intervention. Synovial fluid samples seemed to have more value as potential biomarkers with associations with injury, structural and patient-reported outcomes, however, not all studies had appropriate control samples and therefore it is not fully clear how synovial fluid differs in those with PTOA. The correlation between paired serum and synovial fluid samples was consistently weak, possibly indicating variations in systemic and local concentrations due to some biomarkers being produced locally within the joint tissues and others released into the circulation before diffusing into the synovial fluid, as well as differences in pathophysiological mechanisms [2,67]. Additionally, the rapid and fluctuating proinflammatory expansion of synovial fluid volume may lead to decreases in any locally-produced biomarker concentration, further reducing correlations between synovial and serum spaces.

In this systematic review, all bar one study focussed on ACL injuries, with most undergoing surgical reconstruction, proving homogeneity in pathology. However, many studies did not control for co-existing injuries. This is an area which also requires further exploration, to see if there are other associations to be found with differing aetiologies. The single study with different pathology, combat-related traumatic amputation [40], had the most significant changes in serum biomarkers. It is possible that this could be related to the systemic response following trauma, and the influence of this on PTOA and biomarker concentrations should be explored [8].

Another uncontrolled confounding variable for inflammation, and cartilage/bone metabolism, is the effect of the ACL-R-related trauma and subsequent rehabilitation on the joint remodelling response and associated biomarkers, as demonstrated in the KANON study [37]. These patients may present a higher risk of PTOA, although surgery also has the potential to reduce long-term joint instability.

Limitations of this review include the number, and variable quality, of the studies included. Whilst most studies were satisfactory or good for RoB assessment, individual study limitations weaken the overall results, including small study populations, no appropriately controlled comparison population, and a significant risk of unrecognised bias with study methodology, lack of power and validation in other populations. Significant differences in study methodology prevent too much generalisability and direct comparison between studies. There were no studies measuring plasma or urine biomarkers. Only five biomarkers were performed by multiple studies, all performed by different laboratories, minimising comparability (Table 3). Further limitations apply between studies, such as the variation in reporting methods (such as MRI-scoring, ACLOAS or WORMS) or variation in criteria applied (such as different K-L classification grades employed in different studies). Strengths of this study include the range of databases searched and the inclusion of abstracts that meet inclusion/exclusion criteria (presented in Supplementary File 3) to demonstrate ongoing work in progress and mitigate potential publication bias [41].

In conclusion, whilst the use of biomarkers has the potential to offer insight into the development, progression, and impact of therapy, at present, this review of biomarkers implicated in the chronic phase of PTOA demonstrates that better evidence is required to achieve that. Overall, there is too much heterogeneity to allow direct comparison with differing biomarkers, differing time points, differing assays, and varying qualities of study. There was consensus around ACL-injury as a condition of particular interest and common biomarkers such as those shortlisted by the FDA/OARSI initiative [5,22], although only one of the five biomarkers measured by multiple studies (serum COMP, Table 3) is on that list, highlighting the need for a unified approach as evidence is gathered regarding nascent biomarkers in differing populations. This review did not identify any studies using metabolomics, which may offer another route for PTOA biomarker identification in the future [28,47]. An internationally agreed consensus is required to create recommended guidelines for PTOA research, including standardisation of the biomarker panel assessment, performing ‘time from injury’ sub-analysis, and collection of the same outcome measures, to enable direct study comparisons and meta-analysis in the future.

## Ethics approval and consent to participate

Not applicable.

## Consent for publication

Not applicable.

## Availability of data and materials

Data, including data extraction forms and assessment tools, will be made available upon reasonable request to the corresponding author. All data used in the analysis is provided within the manuscript and supplemental files.

## Funding

This work was supported by a grant from Versus Arthritis (21076) and funding from the UK Ministry of Defence (2122.030). Funders were not involved in the preparation or publication of this work.

## Author contributions

OOS conceived the study. KS and OOS performed the searches. OOS, PL and CH performed the screening, data extraction and bias assessments. OOS drafted the first, and subsequent versions, of the manuscript with feedback from all authors. SK, ANB and AV provided expert guidance throughout. OOS acts as the guarantor for the study.

## Declaration of competing interest

There are no conflicts of interest for any authors.
