# Supplementary material for: Current status of catabolic, anabolic and inflammatory biomarkers associated with structural and symptomatic changes in the chronic phase of post-traumatic knee osteoarthritis– a systematic review
Source: Osteoarthr Cartil Open. 2023 Oct 5;5(4):100412. doi: 10.1016/j.ocarto.2023.100412 (PMC10590857; doi:10.1016/j.ocarto.2023.100412)
Supplement: Multimedia component 1 [file mmc1.docx]

Supplementary Material

Supplementary File 1: Search strategy

Medline search.

| Medline (Ovid MEDLINE® Epub Ahead of Print, In-Process & Other Non-Indexed Citations, Ovid MEDLINE® Daily and Ovid MEDLINE®) 1946 to present | |
| --- | --- |
| ######### | https://ovidsp.ovid.com/ovidweb.cgi?T=JS&NEWS=N&PAGE=main&SHAREDSEARCHID=3buR01lw3SAoYOfW9Xhq1OeJQ7C8egjdJrhNw91qwciJJs0xnI4Vn0zRBkWMLzd5T |
| 1 | Osteoarthritis, Knee/ |
| 2 | (osteoarthr* adj5 knee*).ti,ab,kw. |
| 3 | Osteoarthritis/ |
| 4 | exp Knee/ or exp Knee Joint/ or exp Knee Injuries/ or Arthroplasty, Replacement, Knee/ or Knee Prosthesis/ |
| 5 | knee*.ti,ab,kw. |
| 6 | 4 or 5 |
| 7 | 3 and 6 |
| 8 | 1 or 2 or 7 |
| 9 | ("post trauma*" or post-trauma* or posttrauma*).ti,ab,kw. |
| 10 | (osteoarthrit* adj5 trauma*).ti,ab,kw. |
| 11 | 9 or 10 |
| 12 | exp Biomarkers/ |
| 13 | (biomarker* or biological marker* or biochemical marker*).ti,ab,kw. |
| 14 | serum marker*.ti,ab,kw. |
| 15 | inflammat* marker*.ti,ab,kw. |
| 16 | catabolic marker*.ti,ab,kw. |
| 17 | anabolic marker*.ti,ab,kw. |
| 18 | metabol* marker*.ti,ab,kw. |
| 19 | laboratory marker*.ti,ab,kw. |
| 20 | clinical marker*.ti,ab,kw. |
| 21 | (surrogate endpoint* or surrogate end point*).ti,ab,kw. |
| 22 | Urine/ |
| 23 | exp Blood/ |
| 24 | Synovial Fluid/ |
| 25 | (urine or blood or serum or plasma or synovial fluid).ti,ab,kw. |
| 26 | exp Magnetic Resonance Imaging/ |
| 27 | (magnetic resonance or MRI*).ti,ab,kw. |
| 28 | exp Ultrasonography/ |
| 29 | (ultrasound* or ultrasonograph* or ultrasonic).ti,ab,kw. |
| 30 | imaging.ti,ab,kw. |
| 31 | USS.ti,ab,kw. |
| 32 | or/12-31 |
| 33 | 8 and 11 and 32 |
| 34 | exp animals/ not (exp animals/ and exp humans/) |
| 35 | 33 not 34 |

Embase search

| Embase 1974 to present |  |
| --- | --- |
| 08/11/2022 | <https://ovidsp.ovid.com/ovidweb.cgi?T=JS&NEWS=N&PAGE=main&SHAREDSEARCHID=2OUyggm7SmG5KuAnADFmuCtVLCUS5upMrvRHpqngEPWi64CT3f8HLb4Z5M9ZC1tkS> |
| 1 | knee osteoarthritis/ |
| 2 | (osteoarthr* adj5 knee*).ti,ab,kw. |
| 3 | osteoarthritis/ |
| 4 | exp knee/ or exp knee injury/ |
| 5 | knee*.ti,ab,kw. |
| 6 | 4 or 5 |
| 7 | 3 and 6 |
| 8 | 1 or 2 or 7 |
| 9 | ("post trauma*" or post-trauma* or posttrauma*).ti,ab,kw. |
| 10 | (osteoarthrit* adj5 trauma*).ti,ab,kw. |
| 11 | 9 or 10 |
| 12 | biological marker/ |
| 13 | (biomarker* or biological marker* or biochemical marker*).ti,ab,kw. |
| 14 | serum marker*.ti,ab,kw. |
| 15 | inflammat* marker*.ti,ab,kw. |
| 16 | catabolic marker*.ti,ab,kw. |
| 17 | anabolic marker*.ti,ab,kw. |
| 18 | metabol* marker*.ti,ab,kw. |
| 19 | laboratory marker*.ti,ab,kw. |
| 20 | clinical marker*.ti,ab,kw. |
| 21 | (surrogate endpoint* or surrogate end point*).ti,ab,kw. |
| 22 | urine/ |
| 23 | exp blood/ |
| 24 | synovial fluid/ |
| 25 | (urine or blood or serum or plasma or synovial fluid).ti,ab,kw. |
| 26 | exp nuclear magnetic resonance imaging/ |
| 27 | (magnetic resonance or MRI*).ti,ab,kw. |
| 28 | exp echography/ |
| 29 | (ultrasound* or ultrasonograph* or ultrasonic).ti,ab,kw. |
| 30 | imaging.ti,ab,kw. |
| 31 | USS.ti,ab,kw. |
| 32 | or/12-31 |
| 33 | 8 and 11 and 32 |
| 34 | (exp animal/ or nonhuman/) not exp human/ |
| 35 | 33 not 34 |

Cochrane search

| Date Run: | 08/11/2022 10:03 |
| --- | --- |
|  | <https://www.cochranelibrary.com/advanced-search/search-manager?search=7062384> |
|  |  |
| ID | Search |
| #1 | MeSH descriptor: [Osteoarthritis, Knee] this term only |
| #2 | (osteoarthr* NEAR/4 knee*):ti,ab,kw |
| #3 | MeSH descriptor: [Osteoarthritis] this term only |
| #4 | MeSH descriptor: [Knee] explode all trees |
| #5 | MeSH descriptor: [Knee Joint] explode all trees |
| #6 | MeSH descriptor: [Knee Injuries] explode all trees |
| #7 | MeSH descriptor: [Arthroplasty, Replacement, Knee] explode all trees |
| #8 | knee*:ti,ab,kw |
| #9 | #4 or #5 or #6 or #7 or #8 |
| #10 | #3 and #9 |
| #11 | #1 or #2 or #10 |
| #12 | (post-trauma* or posttrauma*):ti,ab,kw |
| #13 | (osteoarthrit* NEAR/4 trauma*):ti,ab,kw |
| #14 | #12 or #13 |
| #15 | MeSH descriptor: [Biomarkers] explode all trees |
| #16 | (biomarker* or biological NEXT marker* or biochemical NEXT marker*):ti,ab,kw |
| #17 | (serum NEXT marker*):ti,ab,kw |
| #18 | (inflammat* NEXT marker*):ti,ab,kw |
| #19 | (catabolic NEXT marker*):ti,ab,kw |
| #20 | (anabolic NEXT marker*):ti,ab,kw |
| #21 | (metabol* NEXT marker*):ti,ab,kw |
| #22 | (laboratory NEXT marker*):ti,ab,kw |
| #23 | (clinical NEXT marker*):ti,ab,kw |
| #24 | (surrogate NEXT endpoint*):ti,ab,kw |
| #25 | ("surrogate end" NEXT point*):ti,ab,kw |
| #26 | MeSH descriptor: [Urine] this term only |
| #27 | MeSH descriptor: [Blood] explode all trees |
| #28 | MeSH descriptor: [Synovial Fluid] this term only |
| #29 | (urine or blood or serum or plasma or synovial NEXT fluid):ti,ab,kw |
| #30 | MeSH descriptor: [Magnetic Resonance Imaging] explode all trees |
| #31 | (magnetic NEXT resonance or MRI*):ti,ab,kw |
| #32 | MeSH descriptor: [Ultrasonography] explode all trees |
| #33 | (ultrasound* or ultrasonograph* or ultrasonic):ti,ab,kw |
| #34 | imaging:ti,ab,kw |
| #35 | USS:ti,ab,kw |
| #36 | #15 or #16 or #17 or #18 or #19 or #20 or #21 or #22 or #23 or #24 or #25 or #26 or #27 or #28 or #29 or #30 or #31 or #32 or #33 or #34 or #35 |
| #37 | #11 and #14 and #36  Trial registries   \| ClinicalTrials.gov \| <https://clinicaltrials.gov/ct2/results?cond=Post-Traumatic+Osteoarthritis+of+Knee&term=&cntry=&state=&city=&dist=> \| \| --- \| --- \| \| Condition or disease \| Post-traumatic osteoarthritis of knee \| \| Results \| 30 \| \| Searched 8/11/22 \|  \| \|  \|  \| \| WHO ICTRP \|  \| \| Condition \| post-traumatic osteoarthritis \| \| Results \| 50 \| \| Searched 9/11/22 \|  \| |
|  |  |

# Supplementary File 2 - Synthesis without meta-analysis (SWiM) methodology

Grouping studies for synthesis – study description

This review focuses on studies which explored the relationship between traumatic injury and subsequent joint damage using wet biomarkers. All studies used a cross-sectional methodology to measure association, with the KANON-RCT studies also using prospective methodology to test predictive value(1, 2, 3). Study characteristics are described below, with all selected studies reported without further sub-group analysis.

The condition of interest, PTOA, occurs pre-dominantly in a younger population, so to mitigate any confounding from generalised OA, this review only reviewed studies with populations aged between 18 and 45 years old. The average age of participants (reported as both mean and median) was in the late 20’s (1, 2, 3, 4, 5) or early-to-mid 30’s (6, 7) with Zhang reporting their participant age as ‘less than 41’ (8). Female sex confers an increased risk of OA, so this is recorded when reported (all except Zhang(8)). The KANON-RCT participants were 26%(1, 2, 3), Ahlen (4) and Sun 44-45% (5), and Zou (6) 84% female, respectively, whilst Wasser only recruited male participants(7). There were no restrictions on ethnicity in the inclusion/exclusion criteria, with only Sun reporting the ethnic origin of their population (Chinese)(5).

Standard metric and transformation method used – outcome measures used

Six studies measured serum biomarkers, six studies measured synovial fluid biomarkers, and five measured both. All studies used a comparator to measure the biomarker associations, including imaging (all studies used either XR, MRI, or both), PROMS (four studies), histopathology (Zou) and/or biomechanical assessment.

Synthesis methods

Due to the heterogeneity of study design a meta-analysis is not feasible, so a narrative review of the selected study findings has been undertaken.

Criteria to prioritise results

Some studies recognise that their results required validation in a larger population (4, 5), wider demographic (5), over a longer timescale (3), using a prospective design (7) or wider panel of biomarkers (8). Studies from the KANON-RCT have the largest sample size and longest period of observation, however, they acknowledge that they are open to confounding due to lack of adjustment for treatment (surgical/non-surgical management) or cause of injury, biomarker concentrations below the lower level of quantifications and resultant multiple imputation, and unavailable SF samples. Therefore, the results of all studies will be described and discussed, including their specific study limitations. The findings of abstracts which meet inclusion/exclusion criteria are reported to demonstrate ongoing work in progress and mitigate potential publication bias, however, given that these studies have not undergone peer-review, they will not be extensively discussed.

Investigation of heterogeneity

Study findings will be reported in narrative form by biomarker source (serum or synovial fluid), and by associated mechanism (connective tissue turnover and inflammatory response). As all injuries, bar those reported in Wasser,(7) are ACL-related, this will also create homogeneity, as will participant age. However, study methodology (including biomarkers measured and statistical methods), time from injury and sex ratios prevent too much generalisability. Correlation in serum and synovial fluid will be investigated in the studies that paired samples, however, given the limitations of different assay machines and techniques (as explored in Struglics 2018 (1)), this is likely to be assay specific.

Statistical methods employed

Each study used different statistical methodology and modelling to test biomarker association and predictive value. Zhang (8) assessed snoRNA and WORMS associations using Spearman rank correlation and Wilcoxon rank sum tests, with the ability of snoRNA to discriminate WORMS score assessed by ROC curve construction. Ahlen(4) used paired t tests to analyse between injured and non-injured knees, with Wilcoxon signed rank test and Spearman rank correlation to assess the relationship to a comparator. Zou(6) underwent one-way ANOVA or Kruskal-Wallis test to compare differences in SF to different MRI signals. Correlation of sfGhrelin to comparators was performed using Pearson or Spearman correlation analysis and a ROC curve was also completed. Struglics (1) used Pearson correlation to analyse age and COMP levels, correlation between biomarkers at baseline was determined via Kendall’s tau, and longitudinal relationships were explored using mixed regression models with autoregressive covariance structure. Multiple imputations methods were employed for biomarkers below the lower level of quantification. Struglics(3) underwent linear regression to evaluate the relationship between biomarkers and comparators, with a multivariable model assessing all biomarkers, and univariable models assessing them separately, adjusted for age and sex. Multiple imputation was used as before. Roemer(2) developed three predictive models, one with MRI features, one with inflammatory markers, and one which combined the other two, with age and sex included as additional predictors. Model optimization was undertaken to maximise either sensitivity or ROC curve AUC. All biomarker concentrations were log10 transformed prior to analysis. Sun(5) calculated differences between cases and controls with chi-squared, unpaired t- or Mann-Whitney U tests. Kruskal-Wallis tests were performed to compare PACAP differences between MRI groups, with Pearson correlation and multinominal logistic regression analysis performed to assess correlation. ROC curve analysis with AUC was performed. Finally, Wasser(7) used a general linear modal approach to individually assess biomechanical, radioanatomic and biomarker differences between those with and without confirmed OA. Hierarchical regression models were generated to understand contributing factors to joint space narrowing and worsening pain, with Cohen’s d used to determine effect size.

Certainty of evidence

Risk of bias assessments have been performed for all studies using the NOS (Table 3). Studies are graded using a points system; very good 9-10 points, good 7-8, satisfactory 5-6 and unsatisfactory 0-4. The cross-sectional NOS proforma was used in all bar two, when the cohort proforma was more appropriate for the study design. According to this scale, Zhang(8) was rated unsatisfactory; Struglics (2018)(1) Struglics (2020)(3), Roemer(2), Sun(5) satisfactory; and Ahlen(4), Zou(6) and Wasser(7) good.

Study sample sizes ranged from n=11 (4) to n=120/121 (3, 8), with Wasser(7) having n=38, Zou(6) n=61 and Sun(5) n=72 injured participants. Smaller sample sizes increase the risk of Type II error. The use of comparison populations allow confounders to be mitigated, one study had a matched control group, a strength of that study, with three other studies using reference populations (Struglics 2018 used different reference populations for serum and synovial fluid comparison).

Data presentation methods

Data are presented in both narrative synthesis and tabular form, with Table 2 describing study characteristics, Table 3 reporting bias and Table 4 presenting biomarkers performed in more than one study.

Supplementary File 3 – Conference Abstracts

| Author | Year | Title | Journal | Population | Outcome Measures | Key findings |
| --- | --- | --- | --- | --- | --- | --- |
| Nagy et al | 2011 | Serum and synovia MMP-8 in the evaluation of knee osteoarthritis | Osteoporosis International | N=54, 37M;17F. Osteoarthritis  (n=28, 45±3YO), traumatic meniscal injury (n=26, 37±3) | Venous and SF MMP-8, TNFα, IL-6. Subjective scores and IKDC. Cartilage injury evaluation package | High sera & SF MMP8 compared to controls, with some correlations to IKDC. No correlation between sera & SF. Serum TNF correlated to IKDC but not MMP-8. |
| Struglics et al | 2015 | Changes in ARGS-aggrecan, C-terminal type II and nterminal type I collagen telopeptides, and cytokine concentrations over five years after anterior cruciate ligament injury | Osteoarthritis and Cartilage | N=121 acute ACL rupture. Reference healthy population SF serum and urine | SF, serum and urine at baseline (0-6), 16, 30 and 52 weeks, 2 and 5 years post injury. SF and serum IL 6, 8, 10, interferon gamma, TNF-α, aggrecan. Urine CTX-II and NTX-I | SF, not serum, cytokine increased at baseline. At five years, SF TNF-α and urine NTX-I were high and low respectively. Multiple SF, urine and serum biomarkers correlated. |
| Pietrosimone et al. | 2015 | Greater peak vertical ground reaction force and vertical ground reaction force loading rate during walking gait are associated with a lower serum ratio of collagen turnover in individuals with ACL-R | Osteoarthritis and Cartilage | N=19, 8M:11F, 21±2YO, 43±31 months unilateral ACL-R | Resting sera assessed for C2C and CPII. Gait analysis, including stance phase, and loading rate, vGRF | Peak vGRF & vGRF loading rate negatively associated serum C2C:CPII concentration ratio. no association between C2C and CPII to peak, or loading rate of, vGRF. |
| Larsson et al. | 2016 | Prolonged trauma-induced increase of inflammatory cytokines in synovial fluid after surgical reconstruction of ACL ruptures compared to rehabilitation alone: An exploratory analysis in the Kanon trial | Osteoarthritis and Cartilage | N=121, 90M:31F, mean 26, range 18-36YO, acute ACL rupture. 62 immediate ACL-R, 29 delayed ACL-R, 30 rehab only. 21 age-match control | Baseline (0-6 weeks), 4, 8, 12, 24, 60 months post injury. SF available for 63% visits. | Injured cohort had elevated SF cytokine compared to control, reducing with time. Those with ACL-R had higher cytokine levels than rehabilitation |
| Titchenal et al. | 2018 | Mechanically stimulated CS846 correlates with ultrashort echo time enhanced T2* quantitative mri and gait mechanics 2 years after anterior cruciate ligament reconstruction | Osteoarthritis and Cartilage | N=25, 11M:14F, 35±10YO, unilateral ACL-R (2years post-op) | Serum for CS846 (cartilage synthesis) and C1,2C (cartilage degradation) at rest and 6 hours after 30-min treadmill walk, MRI for UTE-T2*, gait analysis including KFM and KAM | CS846 correlated with UTE-T2* and KFM/KAM. No correlation seen with C1,2C. No significant mean difference in CS846 or C1,2C pre/post activity |
| Cameron et al | 2019 | Metabolite profiles discriminate between acl injured cases and uninjured controls within the first year following injury and surgery | Osteoarthritis and Cartilage | N=30, 18M:12F, 19±1YO, ACL injured. N=30 uninjured matched controls. | Plasma underwent mass spectrometry analysis for metabolite profiling | 8 of top 10 metabolites were different in injured cohort at injury, 5 at ACL-R, 6 6-months post-op & 8 at 1 year. |
| Longobardi et al | 2020 | Longitudinal analysis of serum biochemical changes following anterior cruciate ligament injury and reconstruction: A matched comparison-control analysis | Orthopaedic Journal of Sports Medicine | N=34 16M:18F, 21±2YO ACL injury, n=34 16M:18F, 21±2YO uninjured controls | Serum at baseline (within 14 days of injury), 6 and 12 months post-ACL-R for MCP-1, MMP-3, COMP and C2C:CPII | Concentration of MCP-1 & COMP higher in ACL group at all timepoints, no time differences. MMP-3 increased between baseline & 6 months (no other time, inter-group differences), C2C:CPII increased after ACL-R |

M-Male, F-Female, YO-Year Old, SF-synovial fluid, MMP-matrix metalloproteinase, IKDC-, ACL-Anterior Cruciate Ligament, ACL-R-Anterior Cruciate Ligament Reconstruction, IL-interleukin, TNF-tumour necrosis factor, CTX-C-terminal type, NTX-N-terminal type, C2C-collagen type II cleavage product, CPII-collagen type II C-propeptide, vGRF-vertical ground reaction force, UTE-T2*-ultrashort echo time enhanced T2*, KFM-knee flexion moment, KAM-Knee adduction moment, MCP-monocyte chemoattract protein, COMP-cartilage oligomeric matrix protein

Full references

1. Nagy E, Lang E, Csifo E, Gergely I, Zagyva A, Zuh S, et al., editors. SERUM AND SYNOVIA MMP-8 IN THE EVALUATION OF KNEE OSTEOARTHRITIS. OSTEOPOROSIS INTERNATIONAL; 2011: SPRINGER LONDON LTD 236 GRAYS INN RD, 6TH FLOOR, LONDON WC1X 8HL, ENGLAND.
2. Struglics A, Larsson S, Kumahashi N, Frobell R, Lohmander L. Changes in ARGS-aggrecan, C-terminal type II and N-terminal type I collagen telopeptides, and cytokine concentrations over five years after anterior cruciate ligament injury. Osteoarthritis and Cartilage. 2015;23:A52.
3. Pietrosimone B, Blackburn J, Harkey M, Luc B, Pamukoff D, Lane A, et al. Greater peak vertical ground reaction force and vertical ground reaction force loading rate during walking gait are associated with a lower serum ratio of collagen turnover in individuals with anterior cruciate ligament reconstruction. Osteoarthritis and Cartilage. 2015;23:A108-A9.
4. Larsson S, Frobell R, Lohmander L, Struglics A. Prolonged trauma-induced increase of inflammatory cytokines in synovial fluid after surgical reconstruction of anterior cruciate ligament ruptures compared to rehabilitation alone: an exploratory analysis in the kanon trial. Osteoarthritis and Cartilage. 2016;24:S330-S1.
5. Titchenal M, Williams A, Asay J, Migliore E, Erhart-Hledik J, Andriacchi T, et al. Mechanically stimulated CS846 correlates with ultrashort echo time enhanced T2* quantitative MRI and gait mechanics 2 years after anterior cruciate ligament reconstruction. Osteoarthritis and Cartilage. 2018;26:S176-S7.
6. Cameron K, Trump J, Prebihalo S, Svoboda S, Wickiser J, Synovec R. Metabolite profiles discriminate between acl injured cases and uninjured controls within the first year following injury and surgery. Osteoarthritis and Cartilage. 2019;27:S288-S9.
7. Longobardi L, Davis-Wilson H, Pietrosimone B, Creighton RA, Jordan J, Loeser R, et al. Longitudinal analysis of serum biochemical changes following anterior cruciate ligament injury and reconstruction: a matched comparison-control analysis. Orthopaedic Journal of Sports Medicine. 2020;8(7_suppl6):2325967120S00354.

Supplementary File 4 – Excluded Studies

| **Authors** | **Title** | **Journal** | **Year** | **Reason for exclusion** |
| --- | --- | --- | --- | --- |
| Catterall, J. B. et al | Changes in serum and synovial fluid biomarkers after acute injury (NCT00332254) | Arthritis Research and Therapy | 2010 | Study duration |
| Williamson, M. P. et al | 1H nuclear magnetic resonance investigation of synovial fluid components in osteoarthritis, rheumatoid arthritis and traumatic effusions | British Journal of Rheumatology | 1989 | Study duration |
| Lohmander, L. S. et al | Increased levels of proteoglycan fragments in knee joint fluid after injury | Arthritis and Rheumatism | 1989 | Participant age |
| Abe, E. et al | Synovial fluid ferritin in traumatic hemarthrosis, rheumatoid arthritis and osteoarthritis | The Tohoku journal of experimental medicine | 1992 | Participant age |
| Lohmander, L. S. et al | Metalloproteinases, tissue inhibitor, and proteoglycan fragments in knee synovial fluid in human osteoarthritis | Arthritis and Rheumatism | 1993 | Participant age |
| Lohmander, L. S. et al | Stromelysin, tissue inhibitor of metalloproteinases and proteoglycan fragments in human knee joint fluid after injury | Journal of Rheumatology | 1993 | Study duration |
| Lohmander, L. S. et al | Release of cartilage oligomeric matrix protein (COMP) into joint fluid after knee injury and in osteoarthritis | Annals of the Rheumatic Diseases | 1994 | Participant age |
| Lohmander, L. S. et al | Temporal patterns of stromelysin-1, tissue inhibitor, and proteoglycan fragments in human knee joint fluid after injury to the cruciate ligament or meniscus | Journal of Orthopaedic Research | 1994 | Participant age |
| Lohmander, L. S. et al | Increased concentrations of bone sialoprotein in joint fluid after knee injury | Annals of the Rheumatic Diseases | 1996 | Participant age |
| Lohmander, L.S. et al | Procollagen II C-propeptide in joint fluid: Changes in concentration with age, time after knee injury, and osteoarthritis | Journal of Rheumatology | 1996 | Participant age |
| Kuhne, S. A. M. et al | Persistent high serum levels of cartilage oligomeric matrix protein in a subgroup of patients with traumatic knee injury | Rheumatology International | 1998 | Participant age |
| Fang, C. et al | Tissue distribution and measurement of cartilage oligomeric matrix protein in patients with magnetic resonance imaging-detected bone bruises after acute anterior cruciate ligament tears | Journal of Orthopaedic Research | 2001 | Study duration |
| Tourville, T. W. et al | The relationship between markers of articular cartilage metabolism and post-traumatic osteoarthritis following ACL reconstruction | Osteoarthritis and Cartilage | 2011 | Participant age |
| Tourville, T. W. et al | Comparison of synovial fluid ARGS concentrations at baseline and one-year post-ACL reconstruction compared to healthy, matched controls | Osteoarthritis and Cartilage | 2011 | Participant age |
| Tourville, T. W. et al | Relationship between synovial fluid cytokines, MMP's & TIMP's and joint space width narrowing one year post-acl reconstruction | Osteoarthritis and Cartilage | 2011 | Participant age |
| Bigoni, M. et al | Biochemical parameters changes after anterior cruciate ligament (ACL) injury | Journal of Orthopaedics and Traumatology | 2013 | Participant age |
| Tourville, T. W. et al | Relationship between markers of type II collagen metabolism and tibiofemoral joint space width changes after ACL injury and reconstruction | The American journal of sports medicine | 2013 | Participant age |
| Whittaker, J. L. et al | Consequences of knee joint injury in youth sport; implications for osteoarthritis | Osteoarthritis and Cartilage | 2014 | Participant age |
| Whittaker, J. L. et al | Evidence of early post-traumatic osteoarthritis and other negative health outcomes 3-10 years following knee joint injury in youth sport | Osteoarthritis and Cartilage | 2015 | Participant age |
| Pietrosimone, B. et al | Greater Mechanical Loading During Walking Is Associated With Less Collagen Turnover in Individuals With Anterior Cruciate Ligament Reconstruction | The American journal of sports medicine | 2016 | Study duration |
| Furman, B. D. et al | Sphingolipid metabolites are upregulated in human synovial fluid following articular fracture | Journal of Orthopaedic Research | 2017 | Participant age |
| Panina, S. B. et al | Circulating levels of proinflammatory mediators as potential biomarkers of post-traumatic knee osteoarthritis development | Journal of Orthopaedics and Traumatology | 2017 | Participant age |
| Ren, G. et al | A prospective study of serum cytokine profile in individuals at risk of post-traumatic osteoarthritis | Osteoarthritis and Cartilage | 2018 | Participant age |
| Barker, T. et al | Circulating IL-10 is compromised in patients predisposed to developing and in patients with severe knee osteoarthritis | Scientific reports | 2021 | Study duration |
| Manjunath, A. K. et al | Synovial Fluid Biomarkers at the Time of Arthroscopy Predict Five-Year Outcomes | Arthroscopy - Journal of Arthroscopic and Related Surgery | 2021 | Study duration |
| Rodriguez, K. M. et al | The influence of sex and BMI on cartilage metabolism biomarkers in patients after anterior cruciate ligament injury and reconstruction | Journal of athletic training | 2021 | Study duration |
| Markus, D. H. et al | Concentration of synovial fluid biomarkers on the day of anterior cruciate ligament (ACL)-reconstruction predict size and depth of cartilage lesions on 5-year follow-up | Knee surgery, sports traumatology, arthroscopy | 2022 | Study duration |
| Shinmei, M. et al | Significance of the levels of carboxy terminal type II procollagen peptide, chondroitin sulfate isomers, tissue inhibitor of metalloproteinases, and metalloproteinases in osteoarthritis joint fluid | The Journal of rheumatology. Supplement | 1995 | Participant age |
| Pruksakorn, D. et al | Chondroitin sulfate epitope (WF6) and hyaluronic acid as serum markers of cartilage degeneration in patients following anterior cruciate ligament injury | Journal of science and medicine in sport | 2009 | Study duration |
| Wassilew, G.I. et al | The expression of proinflammatory cytokines and matrix metalloproteinases in the synovial membranes of patients with osteoarthritis compared with traumatic knee disorders | Arthroscopy | 2010 | Participant age |
| Jin, W. et al | In vivo H1 MR spectroscopy using 3 Tesla to investigate the metabolic profiles of joint fluids in different types of knee diseases | Journal of applied clinical medical physics | 2016 | Participant age |
| Vnukov, V. V. et al | [PROINFLAMMATORY CYTOKINE GENE POLYMORPHISMS AND THE PLASMA AND SYNOVIAL FLUID LEVELS OF CYTOKINES IN PATIENTS WITH POST-TRAUMATIC KNEE OSTEOARTHRITIS] | Advances in gerontology = Uspekhi gerontologii | 2016 | Language |
| Gao, J. et al. | Reduced miR-519d-3p levels in the synovium and synovial fluid facilitate the progression of post-traumatic osteoarthritis by targeting VEGF | Experimental and Therapeutic Medicine | 2021 | Participant age |
| Turati, M. et al | Characterization of synovial cytokine patterns in bucket-handle and posterior horn meniscal tears | Mediators of Inflammation | 2020 | Participant age |
| Vnukov, V. V. et al | The association between NO-synthase gene polymorphisms and the development of post-traumatic knee osteoarthritis among residents of Rostov region | Russian Journal of Genetics: Applied Research | 2017 | Participant age |
| Neidel, J. et al | Practical aspects of cytokine-determination in the synovial fluid of patients with osteoarthritis, rheumatoid arthritis and other joint-diseases | Zeitschrift fur Orthopadie und Ihre Grenzgebiete | 1996 | Language |
| Luc-Harkey, B. A. et al | Immediate Biochemical Changes After Gait Biofeedback in Individuals With Anterior Cruciate Ligament Reconstruction | Journal of athletic training | 2020 | Participant age |
| Alonso, B. et al | Osteoarthritis-related biomarkers profile in chronic anterior cruciate ligament injured knee | Knee | 2020 | Study duration |

Supplementary File 5 - Correlation between biomarker groups and other variables

Serum

Cartilage turnover

Overall, neither serum COMP, PIIANP or CTX-1 showed a relationship with injury, but serum HA, NTX-1 and C2C did.

Struglics (2018)(1) measured COMP in serum, using two immunoassays (AnaMar, sCOMP-Ana, and BioVender, sCOMP-Bio), which were seen to correlate. BMI was positively correlated with sCOMP-Bio (P=0.001) and sCOMP-Ana (P=0.005). sCOMP-Bio and sCOMP-Ana concentrations were between 1.2- and 1.5-fold higher in men and there was a positive correlation between patient age and sCOMP-Bio and sCOMP-Ana, but no associations were seen with injury. sCOMP-Ana was associated positively with serum ARGS-aggrecan (p<0.001) and sCOMP-Bio was inversely correlated with urine CTX-II at baseline (P=0.04) and inversely associated with urine CTX-II and NTX-1 at five years (P<0.001 and P=0.009, respectively), demonstrating the variability between assays.

Wasser measured collagen II cleavage (C2C, IBEX, Montreal, Quebec, Canada), N-Propeptide of Collagen IIA (PIIANP, MilliporeSigma, Burlington, MA, USA), HA (AbbKine, Wuhan, China), COMP (R&D Systems, Minneapolis, MN, USA); CTX-1 (iDS, Gaithersburg, MD, USA) and NTX-1 (NovusBio, Littleton, CO, USA) via ELISA in 31 of their 38 male traumatic, unilateral lower limb amputation cohort.(7) They saw that those with radiological OA had a 73% lower concentration of HA (P=0.04), 49% lower concentration of NTX-1 (P=0.03) and 44% greater concentration of C2C (P<.01) compared to those who didn’t.

Inflammatory markers

Overall, only serum IL-7 had a strong association to injury, with TNF and IL-10 demonstrating some relationship. Serum IL-1α, 1β, -6, -8, -12p70, -18, IFN-γ, INF-α and TNF-α did not.

Struglics (2020) measured IL-1β, -6, -8, -10, -12p70, IFN-γ and TNF using multiplex Human Pro-inflammatory 7-plex immunoassay (Meso Scale Discovery), with those with more than 20% above LLOQ accepted for analysis (excluding IL-1β) in 116 participants two years after injury.(3) There was no association between molecular biomarkers and outcomes at 5 years, apart from log10 TNF concentration and KOOS pain subscale and serum IL-10 and knee-related quality of life. Roemer used the same markers in 113 of the participants, developing three predictive models based on 2-year data to predict radiographic or MRI OA at the five-year point.(2) The model which focused on inflammatory markers had an AUC of 0.62 and 0.49 respectively. Wasser showed an 180% increase in IL-7 concentration in those with OA compared to those without, and no significant difference in INF-α, IL-1α, IL-18, TNF-α (all ProcarataPlex Human Cytokine/Chemokine kit, Invitrogen, Carlsbad, USA).(7)

Synovial fluid

Cartilage turnover

Overall, in one study synovial fluid COMP showed association to injury (but not the other two studies) and CTX-II limited predictive value, with ARGS-aggrecan and sGAG not.

Ahlen was the only study to analyse SF markers in isolation, measured in 11 participants 8 years post-ACL-R.(4) Biomarker concentrations were compared to clinical assessment, PROMs and imaging, using the non-operated knee as a reference. There was no difference in ARGS-aggrecan (Invitrogen, No. L11XB-1, Meso Scale Discovery), COMP (ELISA, AnaMar Medical AB), and sulphated glycoaminoglycans (sGAG) (Alcain blue precipitation method, Thermo Labsystems) between the operated and non-operated knee.

Zou measured CTX-II and COMP (both R&D Systems Inc., Minneapolis, MN, USA, intra- and interassay CV 7.0% and 7.6%, and 4.8% and 6.8% respectively).(6) AUC analysis showed increased diagnostic value for worsening MRI grades of meniscal injury (Grade 2v1 and 3v2; CTX-II: AUC=0.711 and 0.74; COMP: 0.509 and 0.656, respectively). Struglics (2018) also measured COMP (BioVendor, cat. no.RD194080200).(1) There were no sex differences at baseline. However, over time, levels in men increased to 1.2-1.4 higher, with an initial negative correlation with age settling over the study period. sfCOMP levels remained higher in the initial cohort (2-fold) for up to 5 years (1.4 fold), with the concentration half-life, estimated by random slopes and intercepts analyses, to be 21.9 years (95% CI: 12.4, 95.1). There was no association with serum and sfCOMP (in either assay). sfCOMP was positively associated with synovial fluid cytokine levels (p<0.001), synovial fluid and serum ARGS-aggrecan (p<0.001) and urine CTX-II (p<0.001) and NTX-I (p=0.04). However, there was significant cross-sectional COMP concentration variability between patients at each sampling (CV=31-44%) and longitudinal within-patient variability (CV=21%).

Inflammation markers

Overall, only synovial fluid IL-8 showed weak associations, with IL-1β, -6, -10, TNF, and TNF-α not showing associations or predictive value.

Ahlen also measured IL-1β, -6 and TNF-a via multiplex (K15007C-2; Meso Scale Discovery), with no difference seen between operated and non-operated knees.(4) Struglics (2020) measured IL-6, -8, -10 and TNF using a multiplex Human Pro-inflammatory 7-plex immunoassay (Meso Scale Discovery).(3) No synovial fluid marker measured two years after injury was associated with PROMs at the five-year point. Roemer, using the same markers in the same population, performed a prediction analysis in 78 participants using synovial fluid markers, and a concordance analysis between MRI and synovial fluid markers in 81.(2) The concordance between was weak, with only effusion-synovitis on MRI and sfIL-8 showing a statistically significant relationship. No synovial fluid markers at two years predicted radiographic or MRI-related OA at five years.

Finally, Zou measured IL-6 and TNF-α (both IBL America, Minneapolis, MN, USA; Intra- and interassay CV 4.2% and 7.8% for TNF-α and 5.8% and 6.2% for IL-6) and Sun compared IL-1β and TNF-α (both Cosmobio Co Ltd., Tokyo, Japan; Intra- and interassay CV 2.8-4.1% and 5.5-7.7% for IL-1β, and 3.3-5.9% and 6.1-8.5% for TNF- α) levels.(5, 6) Both performed AUC for MRI meniscal severity grade as before (2v1 and 3v2), with decreasing diagnostic value in worsening meniscal injury and concordance between TNF-α assays, as follows IL-6 (AUC=0.644, P=0.115 and 0.547, P=0.611), TNF-α (0.636, P=0.137 and 0.595, P=0.297), IL-1β (0.675,P=0.03 and 0.584,P=0.304) and TNF-α (0.686,P=0.02 and 0.594,P=0.257).

Specific other markers

Overall, serum U38, synovial fluid ghrelin and PACAP showed associations with injury, with serum ghrelin and PACAP and the other snoRNAs not.

Small nucleolar RNA

Zhang obtained serum from 80 ‘<41 year old’ patients 1 year after ACL-R as part of the Proknee.ca study using Taqman array miRNA profiling and individual RNA assays.(8) They compared results to 60 normal donors without skeletal injury (29 were ‘age-matched within 7 years’ and 31 older). They saw increased serum concentration of snoRNA U24, U38, U48 and U49 in the injured participants. They demonstrated higher concentrations of snoRNA was associated with worse cartilage damage, as defined by WORMS score, however only U38 exceeded statistical significance.

Ghrelin

Zou sought to understand the association of synovial fluid and serum ghrelin (sfGhrelin and sGhrelin) using an LINCO Research radioimmunoassay kit (intra- and interassay CV 9.0% and 13.6%).(6) Their results demonstrated sGhrelin (pg/mL): PTOA, 327.5±84.1; con, 339.8±88.7, and PTOA sfGhrelin 399.5±92.6 (no synovial fluid measured in controls). sfGhrelin was negatively correlated with IL-6, TNF-α, CTX-II and COMP, and severity, measured by arthroscopic Noyes (r=-0.363, P=0.004) and histopathological Mankin score (r=-0.335, P=0.008). sfGherlin was also positively related to Lysholm scores (r=0.405, P=0.001) and IKDC scores (r=0.410, P=0.001). When AUC analysis was performed, sfGhrelin improved with worsening MRI severity (grade 2 vs. grade 1, AUC=0.617, P=0.201; grade 3 vs. grade 2, AUC=0.767, P=0.004). There were no significant differences in sGhrelin between groups, and there was no correlation between sfGhrelin and sGhrelin.

Pituitary adenylate cyclase-activating polypeptide (PACAP)

Sun compared serum and synovial fluid concentrations of PACAP (sPACAP and sfPACAP) by a sandwich ELISA double antibody (Cat No.P18509 ELISA Genie, Dublin, Ireland) against PROMs, MRI meniscal changes and cartilage histology (Mankin scale) in ACL injured individuals.(5) They report lower sfPACAP concentration in the ACL-injured group compared to the control group (202.0 ± 48.3 pg/mL vs326.0 ± 66.7 pg/mL,P< 0.001), with sfPACAP negatively associated with VAS (r=-0.434,P < 0.001), MRI grading (r=-0.537, P < 0.001), and Mankin score (r=-0.417,P < 0.001), and positively associated with Lysholm (r=0.349,P=0.003) and IKDC (r=0.371,P=0.001). Serum PACAP concentrations were not statistically significant between groups (86.0 ± 15.3 pg/mL vs 82.8 ± 19.7 pg/mL, P=0.327). There was no correlation between sPACAP and sfPACAP.

References

1. Struglics A, Larsson S, Pramhed A, Frobell R, Swärd P. Changes in synovial fluid and serum concentrations of cartilage oligomeric matrix protein over 5 years after anterior cruciate ligament rupture: an exploratory analysis in the KANON trial. Osteoarthritis and cartilage. 2018;26(10):1351-8.

2. Roemer FW, Englund M, Turkiewicz A, Struglics A, Guermazi A, Lohmander LS, et al. Molecular and structural biomarkers of inflammation at two years after acute anterior cruciate ligament injury do not predict structural knee osteoarthritis at five years. Arthritis & rheumatology. 2019;71(2):238-43.

3. Struglics A, Turkiewicz A, Larsson S, Lohmander L, Roemer F, Frobell R, et al. Molecular and imaging biomarkers of local inflammation at 2 years after anterior cruciate ligament injury do not associate with patient reported outcomes at 5 years. Osteoarthritis and Cartilage. 2020;28(3):356-62.

4. Åhlén M, Roshani L, Lidén M, Struglics A, Rostgård-Christensen L, Kartus J. Inflammatory cytokines and biomarkers of cartilage metabolism 8 years after anterior cruciate ligament reconstruction: results from operated and contralateral knees. The American journal of sports medicine. 2015;43(6):1460-6.

5. Sun B-Y, Sun Z-P, Pang Z-C, Huang W-T, Wu S-P. Decreased synovial fluid pituitary adenylate cyclase-activating polypeptide (PACAP) levels may reflect disease severity in post-traumatic knee osteoarthritis after anterior cruciate ligament injury. Peptides. 2019;116:22-9.

6. Zou Y-c, Chen L-h, Ye Y-l, Yang G-g, Mao Z, Liu D-d, et al. Attenuated synovial fluid ghrelin levels are linked with cartilage damage, meniscus injury, and clinical symptoms in patients with knee anterior cruciate ligament deficiency. Discovery medicine. 2016;22(123):325-35.

7. Wasser JG, Hendershot BD, Acasio JC, Krupenevich RL, Pruziner AL, Miller RH, et al. A Comprehensive, Multidisciplinary Assessment for Knee Osteoarthritis Following Traumatic Unilateral Lower Limb Loss in Service Members. Military Medicine. 2022.

8. Zhang L, Yang M, Marks P, White L, Hurtig M, Mi Q-S, et al. Serum non-coding RNAs as biomarkers for osteoarthritis progression after ACL injury. Osteoarthritis and cartilage. 2012;20(12):1631-7.
